# Alpha-synuclein seeds in olfactory mucosa and cerebrospinal fluid of patients with dementia with Lewy bodies

**DOI:** 10.1093/braincomms/fcab045

**Published:** 2021-03-22

**Authors:** Daniela Perra, Matilde Bongianni, Giovanni Novi, Francesco Janes, Valentina Bessi, Stefano Capaldi, Luca Sacchetto, Matteo Tagliapietra, Guido Schenone, Silvia Morbelli, Michele Fiorini, Tatiana Cattaruzza, Giulia Mazzon, Christina D Orrù, Mauro Catalan, Paola Polverino, Andrea Bernardini, Gaia Pellitteri, Mariarosa Valente, Claudio Bertolotti, Benedetta Nacmias, Giandomenico Maggiore, Tiziana Cavallaro, Paolo Manganotti, Gianluigi Gigli, Salvatore Monaco, Flavio Nobili, Gianluigi Zanusso

**Affiliations:** Department of Neurosciences, Biomedicine, and Movement Sciences, University of Verona, Policlinico G. B. Rossi, 37134 Verona, Italy; Department of Neurosciences, Biomedicine, and Movement Sciences, University of Verona, Policlinico G. B. Rossi, 37134 Verona, Italy; IRCCS Ospedale Policlinico San Martino, 16132 Genova, Italy; Clinical Neurology Unit, Santa Maria della Misericordia University Hospital, 33100 Udine, Italy; Department of Neuroscience, Psychology, Drug Research and Child Health, 50134 University of Florence, Azienda Ospedaliera-Universitaria Careggi, Florence, Italy; Biocrystallography Laboratory, Department of Biotechnology, University of Verona, 37134 Verona, Italy; Department of Surgical Sciences, Dentistry, Gynecology and Pediatrics, University of Verona, 37134 Verona, Italy; Department of Neurosciences, Biomedicine, and Movement Sciences, University of Verona, Policlinico G. B. Rossi, 37134 Verona, Italy; IRCCS Ospedale Policlinico San Martino, 16132 Genova, Italy; IRCCS Ospedale Policlinico San Martino, 16132 Genova, Italy; Department of Health Science (DISSAL), University of Genova, 16132 Genova, Italy; Department of Neurosciences, Biomedicine, and Movement Sciences, University of Verona, Policlinico G. B. Rossi, 37134 Verona, Italy; Clinical Unit of Neurology, Department of Medicine, Surgery and Health Sciences, Cattinara University Hospital ASUGI, University of Trieste, 34128 Trieste, Italy; Clinical Unit of Neurology, Department of Medicine, Surgery and Health Sciences, Cattinara University Hospital ASUGI, University of Trieste, 34128 Trieste, Italy; Laboratory of Persistent Viral Diseases, Rocky Mountain Laboratories, National Institute for Allergy and Infectious Diseases, National Institutes of Health, Hamilton, 59840 Montana, USA; Clinical Unit of Neurology, Department of Medicine, Surgery and Health Sciences, Cattinara University Hospital ASUGI, University of Trieste, 34128 Trieste, Italy; Clinical Unit of Neurology, Department of Medicine, Surgery and Health Sciences, Cattinara University Hospital ASUGI, University of Trieste, 34128 Trieste, Italy; Clinical Neurology Unit, Santa Maria della Misericordia University Hospital, 33100 Udine, Italy; Clinical Neurology Unit, Santa Maria della Misericordia University Hospital, 33100 Udine, Italy; Clinical Neurology Unit, Santa Maria della Misericordia University Hospital, 33100 Udine, Italy; Clinical Unit of Neurology, Department of Medicine, Surgery and Health Sciences, Cattinara University Hospital ASUGI, University of Trieste, 34128 Trieste, Italy; Department of Neuroscience, Psychology, Drug Research and Child Health, University of Florence, Azienda Ospedaliera- Universitaria Careggi, 50134 Florence, Italy; IRCCS Fondazione Don Carlo Gnocchi, 50143 Florence, Italy; Department of Otorhinolaryngology, Careggi University Hospital, 50134 Florence, Italy; Department of Neurosciences, Biomedicine, and Movement Sciences, University of Verona, Policlinico G. B. Rossi, 37134 Verona, Italy; Clinical Unit of Neurology, Department of Medicine, Surgery and Health Sciences, Cattinara University Hospital ASUGI, University of Trieste, 34128 Trieste, Italy; Clinical Neurology Unit, Santa Maria della Misericordia University Hospital, 33100 Udine, Italy; Department of Neurosciences, Biomedicine, and Movement Sciences, University of Verona, Policlinico G. B. Rossi, 37134 Verona, Italy; IRCCS Ospedale Policlinico San Martino, 16132 Genova, Italy; Department of Neuroscience, Rehabilitation, Ophthalmology, Genetics, Maternal and Child health (DINOGMI), University of Genova, 16132 Genova, Italy; Department of Neurosciences, Biomedicine, and Movement Sciences, University of Verona, Policlinico G. B. Rossi, 37134 Verona, Italy

**Keywords:** dementia with Lewy bodies, alpha-synuclein, real-time quaking-induced conversion assay, olfactory mucosa, cerebrospinal fluid

## Abstract

In patients with suspected dementia with Lewy bodies, the detection of the disease-associated α-synuclein in easily accessible tissues amenable to be collected using minimally invasive procedures remains a major diagnostic challenge. This approach has the potential to take advantage of modern molecular assays for the diagnosis of α–synucleinopathy and, in turn, to optimize the recruitment and selection of patients in clinical trials, using drugs directed at counteracting α-synuclein aggregation. In this study, we explored the diagnostic accuracy of α-synuclein real-time quaking-induced conversion assay by testing olfactory mucosa and CSF in patients with a clinical diagnosis of probable (*n* = 32) or prodromal (*n* = 5) dementia with Lewy bodies or mixed degenerative dementia (dementia with Lewy bodies/Alzheimer’s disease) (*n* = 6). Thirty-eight patients with non-α-synuclein-related neurodegenerative and non-neurodegenerative disorders, including Alzheimer’s disease (*n* = 10), sporadic Creutzfeldt–Jakob disease (*n* = 10), progressive supranuclear palsy (*n* = 8), corticobasal syndrome (*n* = 1), fronto-temporal dementia (*n* = 3) and other neurological conditions (*n* = 6) were also included, as controls. All 81 patients underwent olfactory swabbing while CSF was obtained in 48 participants. At the initial blinded screening of olfactory mucosa samples, 38 out of 81 resulted positive while CSF was positive in 19 samples out of 48 analysed. After unblinding of the results, 27 positive olfactory mucosa were assigned to patients with probable dementia with Lewy bodies, five with prodromal dementia with Lewy bodies and three to patients with mixed dementia, as opposed to three out 38 controls. Corresponding results of CSF testing disclosed 10 out 10 positive samples in patients with probable dementia with Lewy bodies and six out of six with mixed dementia, in addition to three out of 32 for controls. The accuracy among results of real-time quaking-induced conversion assays and clinical diagnoses was 86.4% in the case of olfactory mucosa and 93.8% for CSF. For the first time, we showed that α-synuclein real-time quaking-induced conversion assay detects α-synuclein aggregates in olfactory mucosa of patients with dementia with Lewy bodies and with mixed dementia. Additionally, we provided preliminary evidence that the combined testing of olfactory mucosa and CSF raised the concordance with clinical diagnosis potentially to 100%. Our results suggest that nasal swabbing might be considered as a first-line screening procedure in patients with a diagnosis of suspected dementia with Lewy bodies followed by CSF analysis, as a confirmatory test, when the result in the olfactory mucosa is incongruent with the initial clinical diagnosis.

## Teaser

Perra et al. detected α-synuclein aggregates in olfactory mucosa and CSF samples of patients with dementia with Lewy bodies, using real-time quaking-induced conversion assay. Positivity in unpaired olfactory mucosa and CSF samples was significantly concordant with the clinical diagnosis and nearly 100% in participants in whom paired samples were tested.

## Graphical Abstract

**Graphical Abstract:**
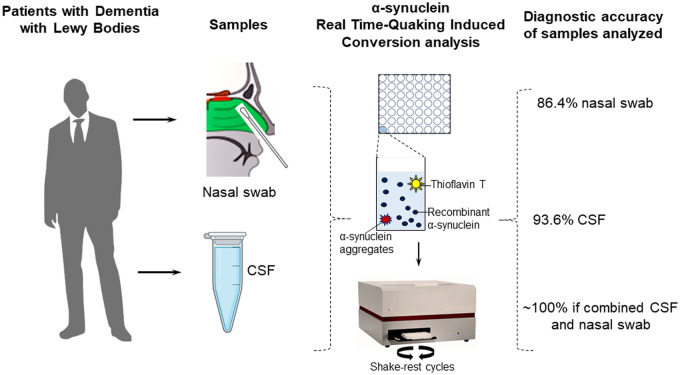

## Introduction

Dementia with Lewy bodies (DLB) is a progressive neurodegenerative disease characterized by fluctuating cognitive decline, visual hallucinations, parkinsonism, and rapid eye movement sleep behaviour disorder. Although the diagnosis of probable DLB is supported by biomarkers of downstream neurodegeneration, such as dopamine transporter SPECT imaging, I-123 metaiodobenzylguanidine myocardial scintigraphy, and polysomnography, the diagnostic accuracy of these tests for DLB is still limited, at early stages of the disease.^1–3^ Moreover, a reliable biomarker for misfolded α-synuclein (α-syn) is demanding. Indeed, a radiotracer for α-syn is not yet available as opposed to Alzheimer’s Disease (AD), in which *in vivo* investigations using radiotracers for beta-amyloid or tau, or CSF determination of biomarker (i.e. phospho-tau, total tau and amyloid β42) levels provides information on the underlying pathology. Despite promising expectations, enzyme-linked immunosorbent assay (ELISA) assays for the detection of disease-associated α-syn in the CSF showed poor specificity and sensitivity.^4^^,^^5^ Therefore, a great interest had been focussed on detecting intraneural phosphorylated α-syn (p-α-syn) deposition by immunohistochemistry in tissue biopsies of skin, colonic submucosa or submandibular glands of patients with DLB for *ante-mortem* diagnosis.^6–8^ However, the diagnostic accuracy of immunohistochemistry in detecting p-α-syn in peripheral tissue biopsies did not provide consistent results among studies, as yet.^9^

More recently, the real-time quaking-induced conversion (RT-QuIC) assay has been shown to reliably detect misfolded α-syn in CSF and other peripheral tissues of patients with α-synucleinopathies.^10^^,^^11^ Alpha-syn RT-QuIC amplifies trace amount of pathological α-syn by virtue of the ability of aggregated α-syn to convert the recombinant α-syn protein, forming amyloid fibrils which enhance fluorescence of thioflavin T (ThT). In a previous study, we tested CSF with RT-QuIC for α-syn in patients with definite and clinical diagnosis of DLB obtaining a diagnostic sensitivity and specificity of 92.9%, and 95.9%, respectively, in distinguishing DLB and DLB with Alzheimer’s co-pathology (DLB/AD mixed dementia) from non α-syn-related dementias.^12^ Similar results in CSF samples from patients with DLB, tested with α-syn RT-QuIC, have been obtained also in other studies.^13–17^

In human prion disorders, we provided evidence that prion RT-QuIC analysis of CSF and olfactory mucosa (OM) samples tested separately provided a 100% specificity and a sensitivity of 77–86% and 97–95%, respectively.^18^^,^^19^ However, the combination of RT-QuIC analysis in both tissues raised the diagnostic accuracy to 100%.^19^ Although in human prion disorders CSF analysis is an urgent and mandatory test to exclude other treatable disorders, in slowly progressive dementias, such as DLB, CSF analysis could be postponed if a minimally invasive procedure such as nasal swabbing would provide the same information.

Alpha-syn RT-QuIC assay on OM samples had been explored in a single study in patients with Parkinson’s disease (PD) or Multi System Atrophy (MSA) versus 18 controls with non-α-syn-related disorders.^20^ More recently, we showed that RT-QuIC assay is positive in OM of 44.4% of patients with iRBD.^21^ However, at present no data are available on α-syn RT-QuIC testing in OM samples from patients with DLB.

In this study, we collected OM samples using nasal swabbing from patients with probable, prodromal DLB and DLB/AD mixed dementia (DLB group) and with non-α-syn-related neurodegenerative or non-neurodegenerative disorders (non-α-syn NDs) and tested them by α-syn RT-QuIC. We also analysed CSF samples to further validate in another specimen the OM RT-QuIC results and to determine the diagnostic accuracy in each of the two tissues and in combination.

## Materials and methods

### Ethics statement and setting

The study was conducted according to the revised Declaration of Helsinki and Good Clinical Practice guidelines. Informed consent was given by study participants or the next of kin.

Five neurology units participated in the study: University of Verona (UNIVR), University of Trieste (UNITS), University of Udine (UNIUD), University of Genova (UNIGE) and University of Florence (UNIFI). OM ad CSF sample collections were performed according to protocols approved by the Ethical Committees of each neurology unit. Written informed consent for OM brushing and lumbar puncture was obtained from each patient or from legal representative.

### Patients

Eighty-one patients were enrolled in the study UNIVR (*n* = 32), UNITS (*n* = 10), UNIUD (*n* = 10), UNIGE (*n* = 22), and UNIFI (*n* = 7) between Jan 2019 and July 2020. Clinical diagnosis was based on the established criteria. OM samples obtained from 43 patients with probable (*n* = 32) or prodromal DLB (*n* = 5) and DLB/AD mixed dementia (*n* = 6) and 38 controls with a clinical diagnosis of non-α-syn NDs, including patients with sporadic Creutzfeldt–Jakob disease (sCJD) (*n* = 10), AD (*n* = 10), progressive supranuclear palsy (PSP) (*n* = 8), corticobasal syndrome (CBS) (*n* = 1), fronto-temporal dementia (FTD) (*n* = 3) (two with behavioural variant and one with non-fluent aphasia) and other neurological disorders (*n* = 6), including, psychosis (*n* = 2), Down syndrome (*n* = 1), vascular dementia (*n* = 1); mild cognitive impairment (*n* = 1) and Arnold–Chiari malformation type 1 (*n* = 1). In particular, the diagnosis of DLB follows the 2017 McKeith criteria^22^ or the 2020 criteria for prodromal DLB.^23^ Patients with DLB/AD mixed dementia included DLB patients with a CSF biomarker profile of AD.^24^ The diagnosis of AD was made according to the 2011 NIA-AA criteria for the dementia stage.^25^ PSP and CBS diagnoses were assessed according to the established diagnostic criteria.^26^^,^^27^ Demographic and main clinical details are reported in Table 1. All patients underwent pertinent brain imaging, as well other investigations supporting the clinical diagnosis and to rule out other causes of cognitive impairment.

**Table 1:** Demographic data of patients with DLB or AD/DLB and non-DLB following the disclosure of clinical data

	Final clinical diagnosis								
	Probable DLB (*n* = 32)	Prodromal DLB (*n* = 5)	DLB/AD (*n* = 6)	AD (*n* = 10)	**Probable CJD** ^a^ **(*n* = 10)**	PSP (*n* = 8)	CBS (*n* = 1)	FTD (*n* = 3)	**Others** ^b^ **(*n* = 6)**
**Gender M/F**	23/9	3/2	3/3	7/3	5/5	4/4	0/1	3/0	2/4
**Age (years)**	73.9 ± 6.0	75.4 ± 4.0	66.3 ± 9.6	69.3 ± 9.6	66 ± 16	73.5 ± 5.0	70	72.0 ± 1.6	53 ± 24.4
**MMSE score at diagnosis**	23.2 ± 4.5	28.0 ± 1.6	18.3 ± 7.4	23.9 ± 1.4	N.E.	27.6 ± 2.9	28	23.3 ± 1.0	27.2 ± 4.2
**Interval between clinical onset and OM swabbing (mo)**	39.0 ± 27.2	26.2 ± 14.9	31.8 ± 31.0	26.0 ± 15.0	3.3 ± 1.8	20.7 ± 14.9	36	37.3 ± 9.6	33.6 ± 43
**MMSE score at OM brushing**	19.7 ± 5.6	27.8 ± 1.5	16.2 ± 8.0	22.7 ± 3.0	N.E.	25.6 ± 4.6	30	16.7 ± 5.0	26.5 ± 3.7
**Interval between clinical onset and lumbar puncture (mo)**	25.6 ± 27.4 (*n* = 10)	24 (*n* = 1)	17.7 ± 12.6 (*n* = 6)	23.8 ± 14.2 (*n* = 10)	3.3 ± 1.8 (*n* = 10)	22.4 ± 17.1 (*n* = 5)	30	31.3 ± 12.7 (*n* = 3)	18.7 ± 6.2 (*n* = 4)
**Alzheimer’s disease CSF profile** ^§^	0/10	0/1	5/5	9/10	0/10	0/4	0	0/3	0/4

a All CJD patients received a definite diagnosis.

b Clinical diagnoses included: Psychosis (*n* = 2), Down syndrome (*n* = 1), vascular dementia (*n* = 1); mild cognitive impairment (*n* = 1) and Arnold-Chiari malformation type 1 (*n* = 1);

§ CSF biomarkers were considered positive for AD. pathology when the ratio of Aβ42 and p-tau was lower than 6.5 (see Ref.^28^) or when the ratio T-tau/Aβ42 was lower than 0.52 (see Ref.^29^); N.E. denotes not evaluated; mo denotes month.

### Study design

OM sample were collected from patients with DLB and from controls with non α-syn NDs in different Neurology units and sent to UNIVR for α-syn RT-QuIC testing. Olfactory mucosa samples from each patient were labelled with an anonymized code and temporary stored at 4°C, until the delivery. Conversely, after collection CSF samples were frozen and sent subsequently. For UNIVR, CSF and OM samples were anonymized by a third party and RT-QuIC analyses were blinded to the clinical diagnosis

### Olfactory mucosa sample collection and processing

Eighty-one patients were included in this study and underwent nasal swabbing. The procedure was well tolerated by all patients and no adverse events were reported. Nasal swabbing procedure was performed in all participants using flocked swabs (FLOQSwabs^®^; Copangroup, Brescia, Italy), as described previously.^19^ Otolaryngologists were trained by a tutorial video of the procedure is available at: https://www.youtube.com/watch?v=wYb9W3u6uMY&t=28s. Each nasal swabbing procedure took around 5 min per patients. Exclusion criteria included severe nasal cavity abnormalities, or infections. Coagulation disorder or anticoagulant/antiplatelet drug intake or other medical conditions were not exclusion criteria. One to four OM samples were collected from each individual, depending on the patient’s tolerance for the procedure. To minimize a potential bias in the pre-analytical step, tubes and nasal swabs for nasal swabbing procedure were provided by UNIVR. Preparation, processing and preservation of OM samples were standardized for all groups.

Following nasal swabbing, the swab was immediately immersed in a 5 ml polypropylene tube containing 0.9% saline, sealed, marked with a code and sent without any personal identifiers to UNIVR within 48 h for α-syn RT-QuIC analysis. Upon arrival, cellular material was dissociated from the swab by vortexing the tubes for 1 min at room temperature. Then, the swab was removed from the tube, and cell suspension was pelleted by centrifugation at 2000×*g* at 4°C for 20 min. The supernatant was almost completely removed, and the remaining pellet frozen at −80°C until assayed. Usually, a single swab would provide a cell pellet with an approximate volume of 15 microliters.

### CSF collection and evaluation of biomarkers

Conversely, CSF samples were obtained by lumbar puncture from 48 patients which included 10 patients with probable DLB, 6 patients with DLB/AD mixed dementia and 32 controls with non-α-syn NDs (Table 1). CSF samples were collected in polypropylene tubes and sent frozen to UNIVR for α-syn RT-QuIC analysis. In 44 CSF samples, biomarkers for AD were also determined and included total-tau (T-tau), phosphorylated-tau (p-tau) and Aβ_1–42_ (Aβ42) analyses using commercially available ELISA kits (INNOTEST htau-Ag, INNOTEST p-tau181 and INNOTEST Aβ42). CSF biomarkers were considered positive for AD pathology when the ratio of Aβ42 and p-tau was lower than 6.5 or when the ratio T-tau/Aβ42 was lower than 0.52.^28^^,^^29^

### Expression and purification of recombinant human α-synuclein

Recombinant α-syn was expressed and purified from the periplasmic fraction as reported.^30^ Briefly, wild-type human α-syn cDNA was cloned in the pET-28a plasmid (Novagen) and transformed into *Escherichia coli* BL21 (DE3). Cell cultures (1 L) were grown at 37°C to an OD 600 nm of 0.3–0.4 and the expression was induced with 0.1 mM isopropyl b-d-1-thiogalactopyranoside (IPTG) for 5 h. Cells were collected by centrifugation, re-suspended in 100 ml of osmotic shock buffer (30 mM Tris–HCl pH 7.2, 40% sucrose, 2 mM EDTA) and incubated for 10 min at room temperature. The cells were centrifuged at 12 000 rpm, re-suspended in 90 ml of cold water with 37.5 µl of saturated MgCl_2_ solution and, after 5 min incubation on ice, centrifuged again. The supernatant containing the periplasm proteins was boiled for 15 min and cleared by centrifugation. The soluble fraction, enriched in α-syn was subjected to ammonium sulphate precipitation followed by extensively dialysis against 20 mM Tris–HCl, pH 8.0. Further purification of α-syn was performed by anion exchange chromatography loading the sample on a Q-Sepharose column (GE Healthcare) equilibrated with the same buffer and eluted with a 0–500 mM linear gradient of NaCl. The purity of α-syn was checked by SDS–PAGE. The protein was then dialysed against 10 mM Sodium Phosphate buffer pH 7.4, and stored at −80°C until use.

### Alpha-synuclein RT-QuIC analysis of olfactory mucosa and cerebrospinal fluid

We performed α-syn RT-QuIC assay as reported previously for CSF and OM except where indicated.^12^^,^^21^

For OM seeded reactions, we used 2 μl of diluted OM in 98 µl of Reaction Buffer composed of 100 mmol/l phosphate buffer (pH 8.2), 10 μmol/l ThT, and 0.05 mg/ml human recombinant full length (1–140 aa) α-syn and 37 ± 3 mg of 0.5-mm glass beads (Sigma). The plate was then sealed with a plate sealer film (Nalgene Nunc International) and then incubated at 30°C in a BMG FLUOstar Omega plate reader with cycles of 1 min shaking (200 rpm double orbital) and 14 min rest. ThT fluorescence measurements (450 ± 10 nm excitation and 480 ± 10 nm emission; bottom read) were taken every 45 min.

In the case of CSF seeded reactions, 15 µl of undiluted CSF was added 85 µl of reaction mix composed of 100 mmol/l phosphate buffer (pH 8.2), 10 μmol/l ThT, 0.05 mg/ml human recombinant full length (1–140 aa) α-syn, 0.0075% sodium dodecyl sulphate (SDS) and 37 ± 3 mg of 0.5-mm glass beads (Sigma). The reaction plates were incubated at 30°C in a BMG FLUOstar Omega plate reader with cycles of 1 min shaking (200 rpm double orbital) and 14 min rest. ThT fluorescence measurements (450 ± 10 nm excitation and 480 ± 10 nm emission; bottom read) were taken every 45 min.

The criteria for discriminating positive versus negative RT-QuIC tests of CSF and OM are criteria similar to those previously described for prion RT-QuIC analyses.^18^ Briefly, a ThT fluorescence threshold was calculated as the average fluorescence during the initial 10 h of incubation, plus three standard deviations (SD) for OM samples and 10 SD for CSF samples. A sample was considered positive overall when at least two of four replicate wells crossed this calculated threshold. When only one of the quadruplicates crossed the threshold, the analysis was repeated, and if the data were confirmed the sample was considered as an undetermined negative. All the data were normalized as previously described.^18^ The results of RT-QuIC analysis were communicated to each Neurology unit or to the third party and matched to the clinical diagnosis.

### Statistical analysis

RT-QuIC relative fluorescence responses were analysed and plotted using the software Graphpad Prism 8.3. We compared the mean relative ThT fluorescence and the lag phase responses in OM and CSF samples from DLB patients by either two-tailed unpaired *t*-test or by Mann–Whitney U-test following ascertainment of normal distribution of data by Shapiro–Wilk normality test.

We compared batch-to-batch difference in the mean maximum ThT fluorescence values obtained in OM and CSF from DLB group and non-α-syn NDs patients by Kruskal–Wallis test with Dunn's multiple comparisons after the ascertainment of normal distribution of data by Shapiro–Wilk normality test.

*P* values < 0.05 were considered statistically significant. Agreement between OM, CSF and clinical diagnosis was tested for significant differences by McNemar test, and quantified with Cohen’s kappa (*k*).

### Data availability

Data can be available upon request to the corresponding author.

## Results

### Detection of α-syn seeds by α-syn RT-QuIC assay

To establish the α-syn RT-QuIC assay for OM and CSF we run an initial set of experiments using a small subset of OM and CSF samples from cases of definite DLB and non-α-syn NDs. Seeding reactions were performed by testing different concentrations of human recombinant α-syn (0.01 mg/ml, 0.07 mg/ml, 0.05 mg/ml, 0.03 mg/ml) and we chose 0.05 mg/ml as the optimal concentration for our current protocol. No increase in fluorescence was observed in OM and CSFs from control patients, while fluorescence above threshold occurred within ∼ 25 h for OM and CSF samples, in reactions seeded with DLB samples (Supplementary Fig. 1).

### Blinded RT-QuIC testing of OM and CSF samples from patients with DLB and with non-α-syn NDs

**Figure 1:**
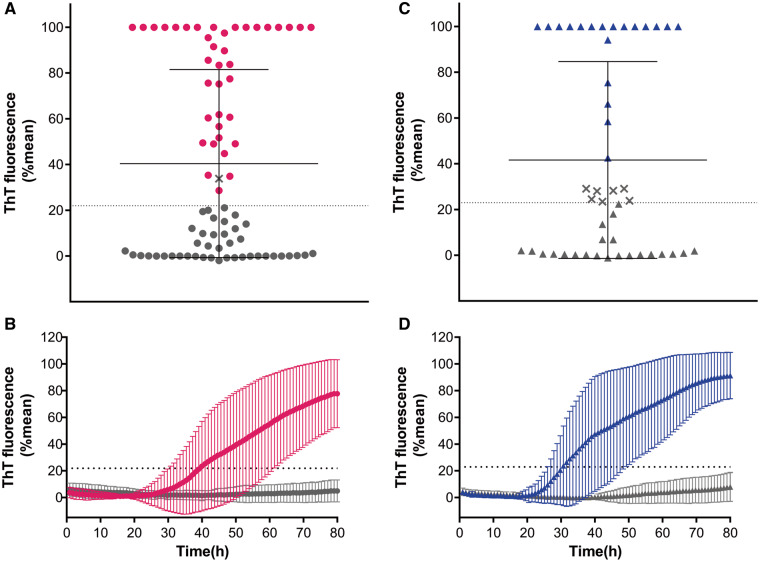
**RT-QuIC blinded analysis of OM and CSF samples from patients with DLB and DLB/AD mixed dementia (DLB group) and with non-α-synuclein-related disorders (non-α-syn NDs).** (**A**) Dots represent the final average relative ThT fluorescence readings obtained in OM for each individual case at 80 h. Bars show the average ± SD for each case. The dashed line shows the fluorescence threshold for a positive result. Magenta dots represent RT-QuIC positive OM while grey dots RT-QuIC negative. The X symbol represents sample with one well positive out of four (**B**) Curves representative of α-syn RT-QuIC from that OM tested RT-QuIC positive (magenta trace) and from OM tested RT-QuIC negative (grey trace). These curves are obtained from the average percentage of Thioflavin T (ThT) fluorescence from four replicate reactions (normalized as described in the Materials and Methods section) with the means (thick lines) of those averages and SDs (thin lines) shown as a function of RT-QuIC reaction time. (**C**) The triangles show the final average relative ThT fluorescence readings obtained for each individual CSF by 80 h. Bars show the average ± SD for type of case. Blue and grey triangles represent RT-QuIC positive and negative CSF samples. The dashed line shows the fluorescence threshold for a positive result. The X symbol represents CSF samples with one well positive out of four. (**D**) Curves representative of α-syn RT-QuIC performed on CSF samples that tested positive (blue trace) and from CSF that tested negative (grey trace). Curves are obtained from the average percentage of ThT fluorescence from four replicate reactions (normalized as described in the Materials and Methods section) with the means (thick lines) of those average and SDs (thin lines) shown as a function of RT-QuIC reaction time.

In a first round, we performed blinded analyses of 81 OM samples. Thirty-nine OM samples gave a positive RT-QuIC response at 30 h while 42 remained negative until 80 h (Fig. 1A and B). Although an OM sample crossed the threshold it did not meet our criteria for RT-QuIC positivity because only one of the quadruplicates and was positive (Fig. 1A).

In a second round, 48 CSF samples were analysed by α-syn RT-QuIC. In 26 samples, the initial seeding reaction was observed at 25 h and the fluorescence gradually increased to 80 h while in 22 CSF samples did not exceed the designated positivity threshold (Fig. 1C and D). Seven CSF samples crossed the threshold but were classified negative, since one well was positive out of four (Fig. 1C).

### Concordance of RT-QuIC results of OM and CSF with clinical diagnosis

**Figure 2:**
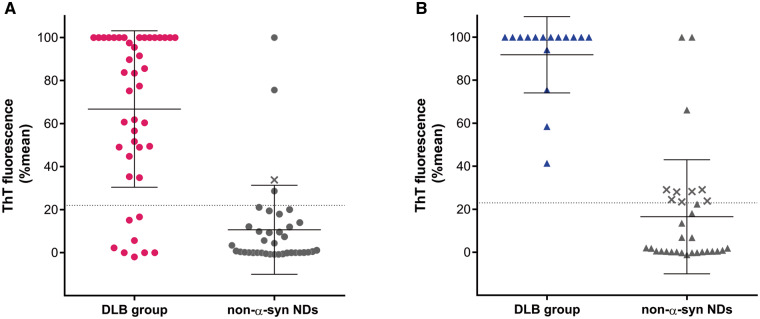
**Unblinded testing by α-synuclein RT-QuIC of OM and CSF samples from patients with DBL and DLB/AD mixed dementia (DLB group) and non-α-syn NDs.** (**A**) Final average relative ThT fluorescence from four replicate readings obtained from OM of each individual cases with DLB group (magenta dots) and for each control (non-α-syn NDs) (grey dots) at 80 h. Bars show the average ± SD for each case. The dashed line shows the fluorescence threshold for a positive result. The X symbols indicate samples which were tested twice and both times had only one well-crossing fluorescence threshold out of the four replicates. These samples were considered as undetermined negative (see Materials and Methods section). (**B**) Final average relative ThT fluorescence from four replicate readings obtained from CSF of DLB group of patients (blue triangles) and for each control (non-α-syn NDs) (grey triangles) at 80 h. Bars show the average ± SD for type of case. The X symbols are considered as undetermined negative.

Once all the OMs and CSFs were tested, the results were unblinded. We learned that 27 RT-QuIC positive OM samples were obtained from patients with probable DLB, five with prodromal DLB and three with DLB/AD mixed dementia while the remaining three positive samples were from patients with non-α-syn NDs (Table 2 and Fig. 2A). These included, one patient with AD, another with atypical parkinsonism and the third with psychosis (Table 2). This first round of RT-QuIC testing showed an accuracy of 86.4% (sensitivity 81.4%, specificity 92.1%). We observed a good agreement between clinical diagnoses and RT-QuIC OM testing (*κ *= 0.729, 95% CI 0.582*–*0.876). The proportion of cases labelled as DLB did not differ between clinical and laboratory diagnosis (*P* = 0.227).

**Table 2:** Results of RT-QuIC assays of olfactory mucosa and cerebrospinal fluid samples

Clinical diagnosis	**Olfactory mucosa** Positive RT-QuIC assay *Number/total number*	**Cerebrospinal fluid** Positive RT-QuIC assay *Number/total number*
**DLB group**	**35/43**	**16/16**
Probable DLB	27/32	10/10
Prodromal DLB	5/5	–
DLB/AD mixed dementia	3/6	6/6
**Non-α-syn NDs**	**3/38**	**3/32**
CJD	0/10	0/10
AD	1/10	1/10
PSP	1/8	1/6
CBS	0/1	0/1
FTD	0/3	1/3
Others	1/6	0/2

Rows in bold text indicate the totals for the DLB group and non-α-syn NDs patients.

RT-QuIC analyses of CSF showed that the 19 out of 47 positive samples belonged to 10 patients with probable DLB and six with DLB/AD mixed dementia while the remaining three were from three patients with a clinical diagnosis of AD, PSP and FTD, respectively (Table 2 and Fig. 2B).

Thus, RT-QuIC was positive in 16 out of 16 CSF samples from DLB group of patients and in three out of 31 from patients with non-α-syn NDs. with an accuracy of 93.8% (sensitivity 100% and specificity 90.6%). We observed a high agreement between clinical diagnosis and RT-QuIC CSF testing (*κ *= 0.866, 95% CI 0.721–1.000), that resulted in a similar proportion of identified cases (*P *=* *0.250).

In the control groups, one OM sample and seven CSF samples had one well out of four replicate reactions with fluorescence exceeding the threshold. These samples did not meet the criteria of positivity and RT-QuIC analysis in these samples was repeated. Upon second testing, the results were the same and thus we considered these samples as “undetermined negative” (Supplementary Fig. 2). The mean curves of fluorescence and the lag phase of these samples showed a progressive increase but below the threshold values within the 80 h timeframe.

### RT-QuIC seeding activity in OM and CSF

**Figure 3:**
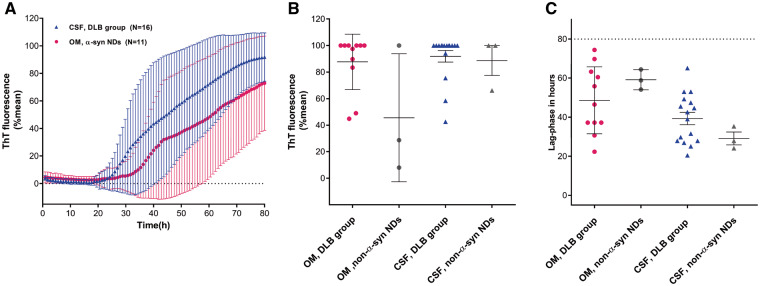
**RT-QuIC detection of α-synuclein seeding activity in patients with DLB and non-α-syn NDs with both OM and CSF samplings.** (**A**) Traces represent the average percentage of thioflavin T (ThT) fluorescence readings from four replicate reactions (normalized as described in the Materials and Methods section), determined in OM (magenta trace) and CSF (blue trace) samples from patients with DLB and DLB/AD mixed dementia (DLB). The means (thick lines) with standard deviations (thin lines) of those averages are shown as a function of RT-QuIC reaction time. (**B, C**) Final fluorescence values in (**B**) and lag-phase in (**C**) of α-syn RT-QuIC positive OM and CSF samples. Samples are grouped in four different classes (OM DLB group, magenta dots, CSF DLB group, blue triangles, OM non-α-syn NDs grey dots and CSF non-α-syn NDs grey triangles). Data points in (**B**), represent the average percent fluorescence value from four replicate readings obtained for each individual case at 80 h and bars show the mean ± SD for type of case. Data points in (**C**) show hours required from the mean percentage of ThT fluorescence value from four replicate readings to exceed the threshold for individual cases and bars show the mean ± SD for type of case.

Then, we determined the relative percentage of maximum fluorescence intensity in “positive” samples obtained in DLB group in OM and CSF. This was approximately 70% for OM and 95% for CSF with a lag phase varying between 30 and 25 h, respectively (Fig. 3). There was not a significant difference in the RT-QuIC average percentage of the final ThT fluorescence value (*P = *0.37 Mann–Whitney test) or lag-time phase (*P = *0.14 *t-*test) between OM and CSF samples from patients with DLB (Fig. 3).

### Batch-to-batch assay reproducibility

We tested OM and CSF samples from cases with definite DLB and with non-α-syn NDs by using three different batches of human recombinant α-syn, as substrate of reaction. After normalization, we did not observe a significant difference in the percentage of maximal ThT fluorescence values, among batches in both CSF and OM samples (Supplementary Fig. 3).

### Evaluation of diagnostic reliability of RT-QuIC results when OM and CSF are combined

In 16 patients and 32 controls, both OM and CSF specimens were available, thus the diagnostic accuracy of RT-QuIC was analysed by combining the results obtained in tissues (Table 3). Five OM samples from DLB group of patients were RT-QuIC negative but the CSFs from the same patients were positive (Table 3). Conversely, three false-positive OMs from patients with non-α-syn NDs had a negative CSF and included one patient with AD, one with mild cognitive impairment and another with CBS. No significant differences between RT-QuIC assay in OM and CSF and clinical diagnoses were observed (*P *=* *0.227) albeit agreement was moderate (*κ*  = 0.498, 95% CI 0.247–0.749).

**Table 3:** Patients with olfactory mucosa only and both olfactory mucosa and cerebrospinal fluid samples analysed by α-syn RT-QuIC

Clinical diagnosis	OM only (33)	Patients with both OM and CSF (16)			
	OM positive	OM and CSF positive	OM positive and CSF negative	OM negative and CSF positive	OM negative and CSF negative
**DLB-Group (43)**	**24/27**	**11/16**	**–**	**5**	**–**
Probable DLB (32)	19/22	8/10	–	2	–
DLB/AD mixed dementia (6)	–	3/6	–	3	–
Prodromal DLB (5)	5/5	–	–	–	–
**Non-α-syn NDs (38)**	**0/6**	**0/32**	**3**	**3**	**26**
CJD (10)	–	0/10	–	–	10
AD (10)	–	0/10	1^a^	1^a^	8
PSP (8)	0/2	0/6	1^a^	1^a^	4
CBS (1)	–	0/1	–	–	1
FTD (3)	–	0/3	–	1	2
Others (6)	0/4	0/2	1	–	1

a This positive sample does not belong to the same patient.

Rows in bold text indicate the totals for the DLB group and non-α-syn NDs patients.

Although the RT-QuIC sensitivity in CSF was 100%, three CSF specimens from patients with non-α-syn NDs tested positive but their OM samples were negative and included one patient with a clinical diagnosis of AD another with PSP and one with FTD (Table 3).

In conclusion, when the results of RT-QuIC in OM and CSF results were combined the diagnostic accuracy increased to 100%.

## Discussion

Clinical diagnosis of DLB is still a challenge and the availability of diagnostic assays that detect pathological α-syn in biological specimens of patients with DLB have relevant clinical and potentially therapeutic implications.^31^ This study aimed at defining the best diagnostic strategy for the identification of pathological α-syn, using the α-syn RT-QuIC assay, in the “optimal diagnostic tissue” such as olfactory mucosa or CSF.

To date, several studies have demonstrated the presence of p-α-syn in skin and lip biopsies from patients with a clinical diagnosis of DLB by immunohistochemistry, showing a sensitivity ranging from 100% to 50% and a specificity from 100% to 97%. However, tissue biopsies are invasive, requiring multiple samplings and the accuracy of immunohistochemistry in detecting p-α-syn is variable among studies.^6–8^ Taking advantages of nasal swabbing, we collected OM samples using this rapid, simple procedure which does not require patient preparation or interruption of anticoagulant therapy and does not damage the olfactory function. Since the olfactory mucosa is approximately 2.5 cm squared and the swab surface is 2 cm in length, the swab comes in contact with a large part of the olfactory epithelium allowing for an efficient OM collection. Therefore, unlike other peripheral tissue biopsies, a successful sampling of OM is always expected.

Of course, RT-QuIC assays have revolutionized the diagnostic approach not only in human prion disorders but also in other proteinopathies and in particular in α-synucleinopathies.^32^^,^^33^ Previous studies showed that α-syn RT-QuIC detects α-syn aggregates in CSF of patients with DLB, with high diagnostic accuracy.^12–17^

Here, we show for the first time that OM samples are α-syn RT-QuIC positive in 35 out of 43 patients with a clinical diagnosis of DLB and in three with non-α-syn NDs with a sensitivity and specificity of 81.4% and 92.1%, respectively. Compared to the previously reported RT-QuIC results in OM of patients with PD and MSA, and iRBD, the RT-QuIC sensitivity in DLB is apparently higher.^20^^,^^21^ This finding might be related to the detection of α-syn deposition in the olfactory neuroepithelium of DLB individuals.^34^

Notably, in all patients with prodromal DLB, OM RT-QuIC resulted positive providing evidence that the process of α-syn seeding aggregation might be revealed at early disease stage. However, given the relative low number of investigated patients, further studies are needed to confirm whether OM swabbing might be considered a reliable diagnostic assay in this group of patients.

Three positive OMs were assigned to patients with non-α-syn NDs but a similar result was also reported in OM from patients with atypical parkinsonism.^20^ Although, the detection of α-syn seeding reaction in OM from patient with AD might argue on the presence of α-syn co-pathology, the positivity in the OM of a relatively young patient with psychosis should be interpreted with caution and thus deserving of clinical follow-up.

We showed that RT-QuIC testing of CSF correctly identifies DLB patients with 100% sensitivity and 90.3% specificity, in line with previous studies.^12–17^ It should be noted that in patients with a clinical diagnosis of DLB/AD mixed dementia and CSF biomarkers with AD profile, the CSF was positive in all cases indicating that α-syn RT-QuIC is a reliable assay for detecting α-syn aggregates.^12^^,^^35^ These findings are in line with our own previous study on α-syn RT-QuIC analysis of 15 CSF samples obtained from pathologically confirmed cases of DLB/AD mixed pathology where we found a positive RT-QuIC for α-syn in 14 CSF analysed.^12^

In both OM and CSF, we found a considerable agreement between clinical diagnoses and RT-QuIC assay. In particular, the sensitivity of CSF was higher compared to OM (100% versus 81.4%) while the specificity was comparable in CSF versus OM (90.3% versus 92.1%). Indeed, the 100% concordant results of RT-QuIC in CSF samples analysis of DLB group argue that positivity in the three controls with AD, PSP and FTD might be related to the presence of coincident α-syn aggregates, as also observed in previous studies.^16^^,^^17^ As such, neuropathologic studies reported a variable frequency of Lewy bodies in cases of AD, PSP and FTLD indicating that the concomitant deposition of α-syn and tau aggregates is a rather common observation.^36^

Our strategy of “double tissue α-syn RT-QuIC testing” from the same patient, by testing of OM first and subsequently CSF, as a confirmatory analysis showed 100% concordance with the clinical diagnosis when both specimens are considered. However, we could perform this kind of combined analysis in approximately half of patients with DLB. In particular, a confirmatory CSF analysis could not be verified in three DLB patients with RT-QuIC negative OM samples because CSF samples were not available.

Of course, lack of both OM and CSF specimens in all patients represents a limitation of the present study and additional investigations on a larger cohort of patients are needed. However, these preliminary data indicate that testing both OM and CSF would be a promising approach to improve the refinement of *intra-vitam* diagnosis in patients with suspected DLB or DLB/AD mixed dementia, with the limitation that these analyses do not dissect Parkinson’s dementia from DLB diagnoses.

In conclusion, we demonstrated that a gentle procedure such as OM swabbing detects α-syn aggregates in OMs with a considerable diagnostic robustness. Conversely, CSF might be considered as an ancillary test to be analysed in the absence of a concordance between clinical diagnosis and RT-QuIC results. Therefore, we envision OM RT-QuIC testing as a technique that could be used to select patients for clinical trials aimed at developing drugs that target α-syn aggregation.^31^

## Supplementary material

Supplementary material is available at *Brain Communications* online.

## Funding

This work was supported in part by Fondazione Cariverona: “Development and validation of a novel molecular assay for alpha-synuclein in patients with Parkinson’s disease and other alpha-synucleinopathies” to G.Z. and Brain Research Foundation Verona. This study was supported in part by the intramural Research Program of the National Institute of Allergy and Infectious Diseases, National Institutes of Health (NIAID/NIH).

## Competing interests

C.D.O. has a patent Provisional (US): 62/567,079 pending, a patent PCT: PCT/US2018/052968 pending, a patent Canada: 3074914 pending, a patent Europe: 18786583.7 pending, and a patent U.S.: 16/652,804 pending. All other authors report no competing interests.

## Supplementary Material

fcab045_Supplementary_Data

## Abbreviations

AD
=
Alzheimer disease
α-syn
=
α-synuclein
CBS
=
corticobasal syndrome
DLB
=
dementia with Lewy bodies
FTD
=
fronto-temporal dementia
MSA
=
multiple system atrophy
NDs =
=
neurodegenerative disorders
OM
=
olfactory mucosa
p-α-syn
=
phosphorylated α-synuclein
PSP
=
progressive supranuclear palsy
REM
=
rapid eye movement
RT-QuIC
=
real-time quaking-induced conversion
ThT
=
thioflavin T
