# Supplementary material for: Alpha-synuclein seeds in olfactory mucosa and cerebrospinal fluid of patients with dementia with Lewy bodies
Source: Brain Commun. 2021 Mar 22;3(2):fcab045. doi: 10.1093/braincomms/fcab045 (PMC8042247; doi:10.1093/braincomms/fcab045)
Supplement: fcab045_Supplementary_Data [file fcab045_supplementary_data.pdf]

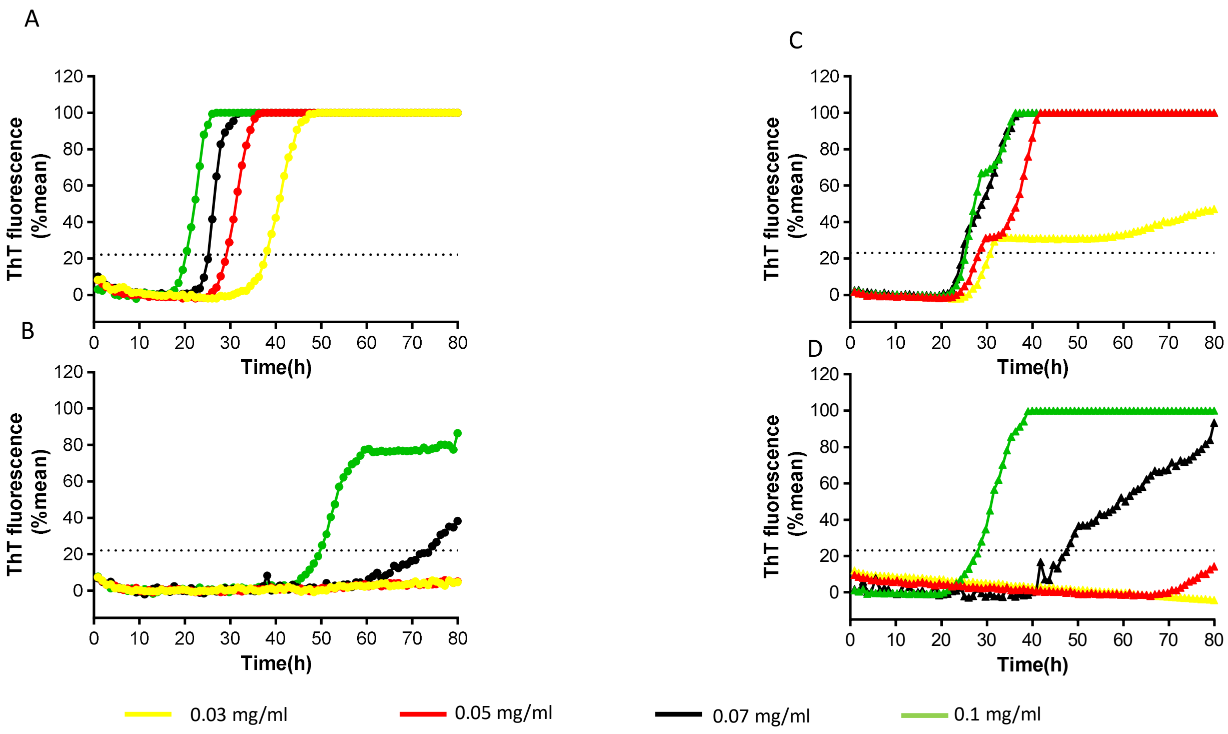

Supplementary Figure 1. Detection of α-syn seeds by α-syn -RT-QuIC assay. Kinetic curves of the OM (dots-line) and CSF (triangles-line) from definite DLB (A-C) and DLB group samples (B-D) tested with different batches of recombinant α-syn. Each color depicts the performance of a different range of concentrations of recombinant human α-syn (0.01mg/ml, 0.07 mg/ml, 0.05 mg/ml, 0.03 mg/ml). Kinetic curves are obtained from the average percentage of ThT fluorescence from four replicate reactions.

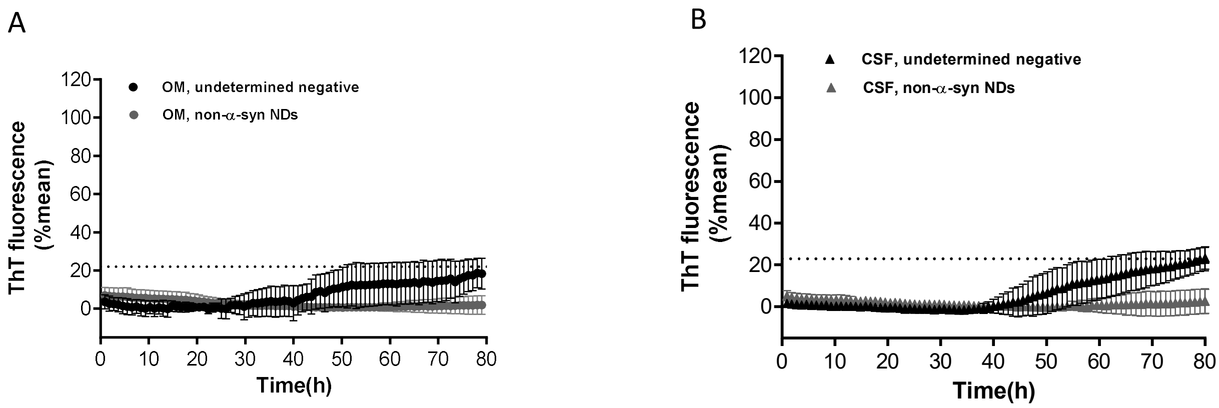

Supplementary Figure 2. Seeding activity of OM and CSF samples "undetermined negative" (see material and methods). Six OM and ten CSF samples with one well positive out of four were repeated albeit they did not all necessarily crossed the threshold. (**A**-**B**) Average percentage of Thioflavin T (ThT) fluorescence from four replicate reactions (normalized as described in the Methods section) with the means (thick lines) and SDs (thin lines) shown as a function of RT-QuIC reaction time. Curves representative of α-syn RT-QuIC from OM (**A**) and CSF (**B**) considered as "undetermined negative" (Black trace) and RT-QuIC true negative non-α-syn NDs (gray trace).

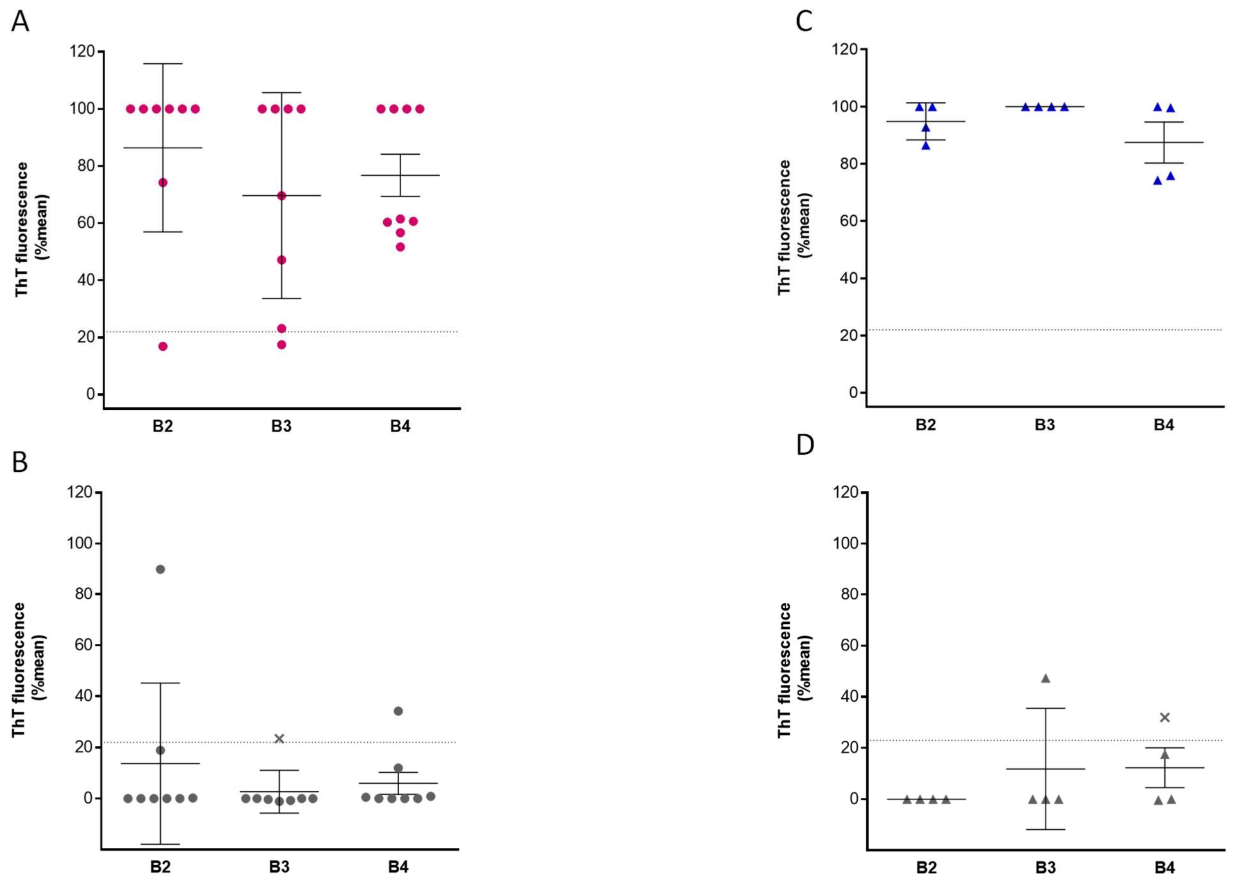

Supplementary Figure 3. Batch-to-batch variation of the maximal ThT fluorescence percentage values obtained in OM and CSF from the same DLB and non-α-syn NDs patients using three different human recombinant αSyn substrate batches. (**A**) Magenta data points show the maximal ThT fluorescence percentage values of quadruplicate wells normalized as described in the methods obtained from OM of a DLB definite case (**B**) and for each control (non-α-syn NDs) (gray dots) at 80 hours. (**C**) Final average relative ThT fluorescence from four replicate readings obtained from CSF of DLB definite case (blue triangles) (**D**) and for each control (non-α-syn NDs) (gray triangles) at 80 hours. Bars show the average +/− SD for type of case. The X symbols are considered as undetermined negative. The dotted line indicates fluorescence positivity threshold.
